# The Role of Targeted Focal Therapy in the Management of Low-Risk Prostate Cancer: Update on Current Challenges

**DOI:** 10.1155/2012/587139

**Published:** 2012-12-31

**Authors:** Daniel W. Smith, Diliana Stoimenova, Khadijah Eid, Al Barqawi

**Affiliations:** Division of Urology, UC Denver School of Medicine, Academic Office One Building, Room 5602, 12631 East 17th Avenue C-319, Aurora, CO 80045, USA

## Abstract

Prostate cancer is one of the most prevalent cancers among men in the United States, second only to nonmelanomatous skin cancer. Since prostate-specific antigen (PSA) testing came into widespread use in the late 1980s, there has been a sharp increase in annual prostate cancer incidence. Cancer-specific mortality, though, is relatively low. The majority of these cancers will not progress to mortal disease, yet most men who are diagnosed opt for treatment as opposed to observation or active surveillance (AS). These men are thus burdened with the morbidities associated with aggressive treatments, commonly incontinence and erectile dysfunction, without receiving a mortality benefit. It is therefore necessary to both continue investigating outcomes associated with AS and to develop less invasive techniques for those who desire treatment but without the significant potential for quality-of-life side effects seen with aggressive modalities. The goals of this paper are to discuss the problems of overdiagnosis and overtreatment since the advent of PSA screening as well as the potential for targeted focal therapy (TFT) to bridge the gap between AS and definitive therapies. Furthermore, patient selection criteria for TFT, costs, side effects, and brachytherapy template-guided three-dimensional mapping biopsies (3DMB) for tumor localization will also be explored.

## 1. Background

Prostate cancer, with an annual incidence of 240,000 new cases in the United States, accounts for 29% of all male cancers [1, 2]. Such a high rate of incidence is attributable to the advent of prostate-specific antigen (PSA) as a screening tool in the 1980s [3]. This screening tool and subsequent treatment led to an initial decrease in prostate cancer mortality until 1993, but this has since leveled off [4]. The reason for this may be the stage migration created by PSA screening. The more aggressive cancers were treated and we are now diagnosing more low-risk disease at clinically lower stages [5, 6]. The recently published PIVOT trial by Wilt et al. investigating prostate cancer mortality with observation (also called active surveillance (AS)) versus radical prostatectomy (RP) defined low-risk prostate cancer as a PSA value ≤10 ng/mL, a Gleason grade of ≤6, and a stage T1a-c or T2a tumor [7]. This includes men who may never progress to fatal disease. While 17% of men will be diagnosed with prostate cancer during their lives, the risk of dying from the disease is only 3% [7].

Along with the increase in diagnosis, we saw a resultant increase in treatment [8]. Treatment modalities included hormone therapies, cryotherapy, targeted focal therapy, brachytherapy and external-beam radiation, and radical prostatectomy, the latter two being utilized most frequently [9]. But while radical prostatectomy has recently been shown to reduce all-cause mortality in those with PSA values >10 ng/mL, no benefit has been shown with values ≤10 ng/mL [7]. Men with low-risk disease are thus receiving all the surgical morbidity associated with more aggressive treatment without any mortality benefit.

To remedy this, a number of advances must take place. Continued research on the benefits and harms of PSA screening as seen in the European Randomized Study of Screening for Prostate Cancer (ERSPC) and Prostate, Lung, Colorectal, and Ovarian (PLCO) Cancer Screening Trial is necessary from an epidemiologic standpoint. Investigations into other potential biomarkers for prostate cancer continues but as of yet has not yielded a better screening tool than the PSA [10, 11]. Further research into potentially better staging techniques such as brachytherapy template-guided three-dimensional mapping biopsies (3DMB) must take place. Finally, treatment options for men who are diagnosed with low-risk tumors and who desire treatment need to be further explored. Modalities that do not possess the significant morbidities associated with the more aggressive options must be vetted and if found to be beneficial, put into more widespread use. These newer techniques must be compared to AS and more definitive treatments in terms of both their efficacies and side effect profiles.

## 2. Overdiagnosis and Overtreatment

Defined by Heijnsdijk et al. as “the detection of prostate cancer during screening that would not have been clinically diagnosed during a man's lifetime in the absence of screening,” overdiagnosis is a major challenge to overcome in the treatment of prostate cancer [12]. Before the advent of PSA as a screening tool patients were often diagnosed at more advanced stages due to the relatively asymptomatic course of the disease [13]. To be sure, more of these individuals can be discovered earlier in their disease progression [5]. The inability, though, to discern which individuals will progress to fatal disease means that a significant number of men with relatively benign behaving disease will receive a cancer diagnosis and may ultimately undergo treatment [5].

Though no PSA cutoff has been shown to yield both high sensitivity and specificity, its use continues as other biomarkers have yet to show better results [10, 11, 14]. Some 20–50% of asymptomatic men are at autopsy found to have prostate cancer and computerized models utilizing the ERSPC data revealed that 10–56% of tumors detected by screening would never lead to clinical symptoms [12]. Furthermore, the United States Preventive Health Task Force gave annual prostate cancer screening a level D recommendation, indicating that “there is moderate or high certainty that this service has no benefit or that the harms outweigh the benefits” [15, 16].

Screening continues, though, and overdiagnosis ensues. This leads to overtreatment. Some studies report overtreatment rates of at least 30% [17, 18]. Further modeling of the ERSPC data showed that per 1,000 men, annual screening between the ages of 55–69 would result in nine fewer prostate cancer deaths and 73 life-years gained over the lifetime of the patients [12]. But at what cost? The same model predicts that there would be 45 cases of overtreatment and that when adjusted for quality-of-life side effects only 56 quality of life years (QALYs) would be gained [12].

## 3. Treatment Options and Outcomes

Until now, treatment for prostate cancer has consisted of active surveillance or more definitive treatments, such as radical prostatectomy, radiation either in external-beam or brachytherapy form, cryotherapy, and targeted focal therapy (TFT) [19]. Radical prostatectomy and external-beam radiation have been the dominant treatment forms and will briefly be discussed [5]. But while many men may receive no mortality benefit from definitive treatment, the unpredictability of the disease course is often too much to bear [5]. Anxiety, depression, and emotional distress are associated with uncertainty in many illnesses including prostate cancer [20]. Compounding the problem is a lack of consensus of what defines progression while on active surveillance [21]. Criteria range from utilizing PSA doubling time to percent core involvement to clinical staging [5, 22–24]. Indeed, only 18.5% of men opt for active surveillance instead of definitive treatment [25].

Definitive treatments, though, are not without risks and morbidities. Sexual dysfunction rates between 20 and 70% and urinary incontinence rates of 15–50% are seen with radical prostatectomy [5]. These side effects are common in other treatment modalities as well, with sexual dysfunction and urinary incontinence occurring in 45% and 2–16%, respectively, of patients receiving external-beam radiation [5]. Moreover, these risks and side effects far outweigh the benefits when definitive treatment is used for low-risk disease. The recently published PIVOT trial has shown not only is there no all-cause or prostate specific mortality benefit with radical prostatectomy versus observation for localized disease, but also there was a nonsignificant increase in mortality associated with radical prostatectomy [7]. The trial further demonstrated that when compared with radical prostatectomy, observed patients had incontinence rates of 6.3% (versus 17.1% with RP) and erectile dysfunction at a rate of 44.1% (81.1% with RP) [7].

## 4. Targeted Focal Therapy

Targeted focal therapy is a potential bridge between active surveillance and the more aggressive treatment modalities for men with localized, low-risk disease. Crawford and Barqawi define TFT as the “complete ablation of all clinically significant cancer foci within the prostate using a minimally invasive technique with preservation of the sphincter, normal gland tissue, and the neurovascular bundles” [19]. Various ablative options exist for TFT including high intensity focused ultrasound (HIFU), cryotherapy, brachytherapy, radiotherapy, and thermotherapy [5]. Our institution utilizes cryotherapy as a medium. Freezing and subsequent tissue thawing causes direct cell injury and at the same time induces an inflammatory response, ultimately resulting in cell death [26]. Developed in the 1960s by Cooper and Lee, the first cryotherapy probes used circulating liquid nitrogen to freeze tissue to −200°C [9]. These initial probes, though, resulted in fistulas, incontinence, and urethral strictures [9]. Technological advancements including the placement of transrectal ultrasound (TRUS) to aid in visualization of ice ball formation, the development of thinner cryoneedles, probe placement through a template grid, and many others have in recent years helped to reduce side effects while at the same time increasing the procedure's effectiveness [27].

Success of the procedure depends upon a number of factors. Accurate diagnosis and staging are essential for patient selection [28]. Imaging in the form of 3DMB can localize tumor foci and help guide cryotherapy probe placement [9]. Though consensus has yet to be reached on the optimal protocol, appropriate followup and monitoring of progression are also necessary to determine when further treatment is warranted.

## 5. Patient Selection

As a potential link between active surveillance and more aggressive therapies, TFT is an attractive option for men with tumors suitable for this therapy who wish to avoid the morbidities associated with more radical therapies [9]. Men who may not tolerate aggressive therapies, such as those with previous pelvic surgery or irradiation, morbid obesity, cardiac disease or irritable bowel disease, are also potential candidates [26, 27]. Additionally, it provides a treatment option for men who may otherwise be candidates for AS but who desire some form of treatment.

Minimal differences emerge when comparing patient selection criteria from various sources, but strict criteria have not yet been defined. Nomura and Mimata suggest primary cryotherapy is appropriate in low-risk patients with clinical staging up to T2a, Gleason grade 6, and a PSA < 10 ng/mL [26]. Crawford and Barqawi, as well as Babaian et al. include organ confined disease up to cT2b, with any Gleason grade and a negative metastatic work-up [19, 27]. Underlying this is the fact that cryotherapy has thus far yielded the best results in those with a PSA < 10 ng/mL [27]. It has also proved effective in those with intermediate disease, though, including a Gleason Grade of ≤7, a PSA between 10 and 20, or clinical T2b staging [27].

According to Crawford and Barqawi, those for whom this treatment is contraindicated include those with severe lower urinary tract symptoms due to BPH, multiple cancer foci, large prostates, and tumor foci near the urethra or neurovascular bundles unless potency is not a concern [19]. Nomura and Mimata add that previous TURP is also considered a contraindication [26].

## 6. Imaging and Mapping

Accurate localization of tumor foci is necessary to determine the extent of prostate cancer in a potential TFT candidate, and mapping biopsies can detail the precise location of the cancer [9]. Cadaveric studies utilizing 3DMB to localize prostate cancer have in the past shown better accuracy than sextant biopsies [29]. In addition to helping localize tumor foci for TFT, 3DMB is also warranted when TRUS biopsies are repeatedly negative in the face of a high PSA, an abnormal DRE, or a rapid PSA doubling time [19]. Both a transperineal and a transrectal approach have been described; our institution utilizes the transperineal method as the apical and anterior portions of the prostate are more readily accessible [30]. The transperineal approach may also reduce the risk of rectal bleeding and sepsis [30]. Samples are taken at 5 mm increments along a brachytherapy grid to create a three-dimensional map [9].

Though 3DMB can better localize tumor foci than TRUS biopsy, the method is not without difficulties. Prostates larger than 60 cc may require both transperineal and transrectal biopsies [19]. Short-term 5-alpha reductase inhibitor therapy can be used to shrink the prostate to a size that necessitates only a transperineal biopsy [19]. Additionally, tumors are often located in the periphery of the prostate which is better accessed via a transrectal approach [9]. Regardless, the benefits of 3DMB outweigh the difficulties in obtaining specimens from these selected groups. Barqawi et al. showed that when compared with previous TRUS results in 215 patients, new foci were found in 82 patients and higher Gleason scores were noted in 49 using 3DMB [31]. Another study revealed that in patients with unilateral prostate cancer diagnosed by TRUS biopsy, 61.1% had in fact bilateral disease and 22.7% received higher Gleason grades [32].

## 7. Outcomes/Cost

As TFT continues to evolve, so too do its follow-up protocols. While serial PSA measurements can reveal biochemical failure, agreed-upon schedules have not yet been elucidated nor have definitions of disease progression [19, 27, 33]. The American Society for Therapeutic Radiology and Oncology (ASTRO) uses as a measure of progression three consecutive PSA increases following the posttreatment nadir [26]. The Phoenix criteria define it as PSA nadir plus 2 [26]. To be considered is the fact that PSA measurements are a less reliable form of followup when large portions of the prostate remain [34, 35]. In a 5-year study at our institution looking at cryoablative TFT used in conjunction with 3DMB, TRUS biopsies are performed at one year to evaluate disease progression in addition to serial PSA monitoring.

As a relatively new form of treatment, studies documenting treatment results and costs are continually emerging. A group of studies looking at various forms of cryoablation including targeted focal therapy, hemiablation, and radical ablation found positive biopsies at 6 and 12 months ranging between 7.7% and 23% [36–39]. Studies investigating whole gland cryotherapy found negative biopsy rates between 87% to 98% within the same time frame [27]. Further data from the Cryo On-Line Data (COLD) Registry revealed 5-year disease free rates between 77.6% and 82.4% according to ASTRO criteria and from 58.0% to 74.9% according to Phoenix criteria [26]. An added benefit of this form of treatment is that should the tumor(s) recur, retreatment is possible [19]. And while direct surgical costs are higher ($5,702) with cryosurgery when compared to radical ($2,788) or robotic ($3,441) prostatectomy, the overall cost of the entire hospital stay is less [40]. Hospitalization time is decreased with cryotherapy and the overall cost ($9,195) is less than that for either radical ($10,704) or robotic ($10,047) prostatectomy [40]. Driving costs down further is the understanding that patients do not often require blood transfusions with cryotherapy nor is a pathologic evaluation completed [40].

With targeted focal therapy, disease treatment becomes possible while minimizing side effects seen with more aggressive and invasive forms. With cryohemiablation, impotency rates range from 10 to 29% and normal function can take up to one year to return [33, 41]. Compared with the 20–70% impotency rate seen in radical prostatectomy, TFT is a welcomed arrival [5]. Incontinence rates are also decreased with cryoablation to rates ranging from 3.7% to 4.8% compared to 17.1% with radical prostatectomy noted in the PIVOT trial [7, 42–44].

## 8. Moving Forward

Since their development in the 1990s, focal therapies have shown increasing efficacies without the morbidities associated with the more aggressive therapies [5]. These therapies will likely play an increasing role in the treatment of low-risk prostate cancer as studies such as the PIVOT trial fail to demonstrate a mortality benefit with more aggressive therapies. Thus, continued research and investment into these modalities need to be a focal point of urologic oncology in the coming years.

As a relatively new treatment modality, it has been suggested that this form of subtotal cryotherapy requires further study before recommendations can be made as to its efficacy as a treatment for prostate cancer [27]. Though there are data regarding disease free rates at 5 years with cryotherapy, lengthier mortality studies are needed. How TFT ultimately compares with AS in terms of mortality and disease free progression will prove pivotal when counseling patients on various treatment options. Additionally, consensus definitions of disease progression and follow-up protocols need to be reached. Better staging and tumor localization with newer imaging and biopsy techniques such as 3DMB will hopefully play a significant role in increasing the efficacy of TFT [45]. We look forward to the upcoming publication of results of our 5-year study investigating TFT used in conjunction with 3DMB. We cannot continue to treat low-risk disease with aggressive treatments and their associated morbidities without mortality benefits, and TFT may offer a good solution.
